# College Session

**Published:** 1853-11

**Authors:** 


The Present College Session.—The present session of the "College of Physicians and Surgeons" opened on the 20th ult., under circumstances most flattering and encouraging. A larger class than usual had assembled, since which up to the present writing, others have been almost daily arriving, and several others from whom we have heard are yet to come, Many of those composing the class are already well advanced and have been in practice for some years past, and have enjoyed the confidence and esteem of those among whom they have arduously labored. Actuated by a noble desire for further improvement, they have, for a time, abandoned their several lucrative fields of practice and are now here among us, exhibiting by their devotion to study and acquirement, a determination to make themselves proficients in medical science. So also of the entire class, and we do not hesitate to say that a more intellectual class, for the number, cannot be found. This fact will be most gratifying to the true friends of the school, as it is to ourselves and the profession of the State. To the different officers of the government who have heen so industriously interesting themselves to promote the well-being of this scientific enterprise in our State, the thanks of the profession and the Faculty of the College are respectfully due. Many of the members of the Legislature are themselves physicians, and these have been especially energetic and active, and of disinterested efforts and friendship it will be our pleasure to speak hereafter. To all, officers of the State, impartial medical men, and citizens, we tender an invitation, when in the city during the session, to call and examine for themselves in order that they may be assured how entirely the institution can fulfil their expectations and justify their hopes and wishes for the future. This we ask in the fullest confidence and assurances of the most entire satisfaction.

